# Does multisensory stimulation with virtual reality (VR) and smell improve learning? An educational experience in recall and creativity

**DOI:** 10.3389/fpsyg.2023.1176697

**Published:** 2023-06-15

**Authors:** Veneta Andonova, Felipe Reinoso-Carvalho, Manuel Arturo Jimenez Ramirez, David Carrasquilla

**Affiliations:** Universidad de los Andes School of Management, Bogotá, Colombia

**Keywords:** virtual reality (VR), multisensory stimulation, multisensory learning, recall, creativity

## Abstract

**Purpose:**

The purpose of this paper is to derive into practical recommendations from multisensory stimulation with virtual reality (VR) and scent to help educators develop effective teaching strategies geared toward aspects of the learning experience, recall, and creativity in a stereotypical learning context.

**Design/methodology/approach:**

The paper is based on a randomized experiment in which student participants were subdivided into three treatment groups and one control group. Each group was stimulated by a different combination of visual, auditory, and olfactory stimuli (2D SMELL, VR, and VR SMELL) and the outcomes were compared against those of the control group (2D). Consistent with the Cognitive Theory of Multimedia Learning, hypotheses were constructed to study the effect of different combinations of stimuli on the learning experience and learning outcomes related to recall and creativity in a stereotypical learning context.

**Findings:**

Traditional video content alone and bundled with a coherent olfactory stimulus prompted higher self-reported ratings of perceived quality of the sensory experience. Olfactory stimulus in combination with either VR or a traditional video prompted higher self-reported ratings on perceived immersion. In a stereotypical learning context, the highest recall scores were achieved with traditional video alone. Both VR alone and bundled with an olfactory stimulus resulted in enhanced creativity.

**Research limitations/implications:**

The findings of this study should be interpreted in the context of adopting multisensory stimulations combined with VR technology as part of stereotypical learning contexts. Most professional educators do not have robust knowledge or experience in using build-on-purpose multisensory stimuli but are increasingly engaged in using multisensory tools such as VR, as part of their teaching practice. In relation to recall, the results are consistent with the hypothesis that in a stereotypical learning context, a multisensory experience involving VR and olfactory stimuli can be related to an undesired cognitive load for learners. There exists a possibility that the low-technical version of the VR goggles used, as well as the contents of the instructional video may have influenced the learning outcomes in terms of recall. Hence, future research should consider such aspects and focus on richer learning contexts.

**Originality/value:**

This work offers practical recommendations for instructional design strategies aiming to create multisensory stimulations with VR and olfactory components to foster a richer learning experience and enhanced learning outcomes, under the assumptions of a stereotypical learning context.

## 1. Introduction

Rich multisensory technology, including virtual reality, is becoming more accessible and thus more widely available in all types of educational environments (Weller, 2007; Hanson and Shelton, 2008; Vergara et al., 2019; Parmaxi, 2020). For instance, we can currently access virtual/augmented experiences through videos in 3D format via accessible streaming platforms, such as YouTube. With a pair of headphones and different versions of VR goggles, such content can prompt augmented sensations in users. But can this help to enhance learning outcomes? VR immersive experiences are calling the attention and curiosity of professionals and organizations to engage in experimentation since today’s technological tools promise to upskill and reskill, as urgently required in this Fourth Industrial Revolution (Deloitte Insights, 2020).

Multisensory environments, initially proposed via studies of individuals with cognitive and behavioral impairments (Cleland and Clark, 1966), provided encouraging early outcomes resulting in a cumulative volume of research reporting the broad-ranging benefits of multisensory learning, thus giving rise to the field of multisensory learning as such. The underlying proposition behind it is that the human brain has evolved to develop, learn, and operate optimally in multisensory environments, suggesting that multisensory training better approximates natural settings and, consequently, is more effective for learning (Shams and Seitz, 2008). The Cognitive Theory of Multimedia Learning (Mayer, 2005) builds on these premises and proposes a mechanism of multimedia learning based on three cognitive science principles of learning. Based on theoretical grounds, experiments involving the stimulation of all senses are actively researched in the growing field of VR, since experts anticipate opportunities for significant improvements in teaching and learning through immersive settings (see for example, Kapralos et al., 2017; Lécuyer, 2017). VR is a rapidly improving tool and researchers argue that fully integrating the other senses—beyond vision and audition—into VR, is just a matter of time (Kilteni et al., 2012; Flavián et al., 2021). Technically, the biggest challenge for VR today involves the inclusion of olfaction and taste, due to the chemical basis of these two kinds of sensory inputs. Nevertheless, prominent researchers in the field of digitalization of olfaction (Purdy et al., 2021) and taste (Cheok and Karunanayaka, 2018) suggest that multisensory VR will eventually involve the five senses. In fact, a recent review shows that 84.8 percent of existing studies in the field report some kind of benefit of multisensory VR experiences (Melo et al., 2020). The latter review also suggests that smell is still a sense that is underexplored in multisensory VR experiences.

In the present study, we draw on the recent and growing adoption of VR as a tool that creates immersive multisensory educational environments in which learners can be subjected to stimuli that arguably foster more satisfactory experiences and improved learning outcomes. Specifically, we disentangle the multiple dimensions of the learning experience and separate them from the learning outcomes related to recall and creativity. In a stereotypical learning context, we conjecture that a multisensory VR experience could be generally perceived as a qualitative upgrade, as compared to the stereotypical 2D video, prompting perceptions that would represent a more immersed experience. Indeed, one of the key drivers for effective learning is the ability to retain and, consequently, retrieve information (recall) and use it in new and creative ways (creativity). By applying the Cognitive Theory of Multimedia Learning in a stereotypical learning context, we expect VR-enabled multisensory instruction to be associated with improved recall and enhanced creativity.

To this end, we conducted a randomized experiment in which participants were immersed in an audio-visual 360 degrees stimulation, while in certain conditions olfaction was additionally stimulated in a physical and congruent way. Learners were randomly subdivided into four groups (3D audiovisual stimulus with VR goggles—namely VR; VR plus congruent scent—namely VR SMELL; 2D audiovisual stimulus plus congruent scent—namely 2D SMELL; only 2D audiovisual stimulus—namely control). In sum, each augmented group was stimulated by a different combination of visual and olfactory stimuli (2D SMELL, VR, and VR SMELL), and the outcomes were compared against the control group (2D). We found that traditional video content alone (2D) and bundled with a coherent olfactory stimulus (2D SMELL), prompted higher self-reported ratings of perceived quality of the sensory experience. Olfactory stimulus in combination with either VR (VR SMELL) or a traditional video (2D SMELL) prompted higher self-reported ratings on perceived immersion. In a stereotypical learning context, the highest recall scores were achieved with traditional video alone (2D). Both VR alone (VR) and bundled with an olfactory stimulus (VR SMELL) resulted in enhanced creativity.

The rest of the paper is organized as follows. First, we introduce the basic concepts and the theoretical framework guiding this work. We start by presenting the key traits of VR experiences in the context of educational environments. Then, we discuss multisensory stimulation and its benefits. An important trait of VR experiences is the creation of immersive learning environments to help learners perceive sensations and interact with aspects of the VR experiences of the world by touching, smelling, seeing, tasting, and hearing elements that are not physically present but imagined by the learner as if they were completely real to the point of tinkering with those elements or even reshaping them. Spanning tenets of the Cognitive Theory of Multimedia Learning to the context of a stereotypical learning context, we advance a series of research questions and associated hypotheses regarding the learning experience and the learning outcomes related to recall and creativity. Next, we describe the experimental design, data, analysis, and outcomes. Finally, we discuss the limitations and implications of the study along with avenues for future research.

### 1.1. VR experiences, senses, and the cognitive theory of multimedia learning

Broadly defined, a VR experience is a computer-based technology that allows users to immerse themselves in an interactive experience or world (Zheng et al., 1998; Ryan, 1999). With the aid of computer graphics, VR experiences enables engineers, graphic designers, educators, and other technically experienced professionals, by enabling a simulation that creates realistic virtual worlds that can be manipulated by users –people who immerse themselves in such VR worlds—in real time. These users can interact with the virtual worlds through different types of direct inputs such as verbal commands, gestures, movement, etc. This is achieved through multiple sensorial channels that include mainly visual, auditory, but also progressively tactile, olfactory, and even simulations of taste sensory modalities (Burdea and Coiffet, 2003). These interactions (namely inputs) are detected by the computer responsible for the VR architecture and can immediately alter the virtual world according to what the user is doing, e.g., the user can grab an object such as an apple in the VR world and move it to a new position. Once the user moves, in this case the apple, the virtual world will immediately detect the movement and produce the simulation of that apple in the position to which it has been moved (Burdea, 1996).

There are multiple frameworks that help highlight the differentiating aspects of VR when compared to other technologies. For example, according to Burdea and Coiffet (2003), VR has three key features that separate it from other types of worlds such as the usual audiovisual video presented in two dimensions. These are: (a) interactivity (Weiner and Simpson, 1989), (b) immersion, and (c) imagination. VR can also be categorized depending on the level of interaction, the levels of immersive environments, and/or the levels of augmented reality. Concerning the latter, many immersive environments are not fully immersive, but augmented. This means that the user is partially immersed in a virtual world by using a computer or a smartphone, interacting with the presented material via a mouse, keyboard, joystick or touchscreen, amongst other tools, and aided by VR goggles, which may be accompanied by headphones (Lee and Wong, 2008). Such differentiation characteristics make VR suitable to be used in multiple professional fields, such as movie production (Lee and Kim, 2016), marketing (Alcañiz et al., 2019), psychology therapy and treatments for conditions such as anxiety disorders (Difede et al., 2007; Powers and Emmelkamp, 2008; Pallavicini et al., 2016), as well as for product design, military and aerospace, entertainment, education and training (Liu et al., 2018).

In the context of education and training, VR has been used as a tool both for children and adults. That is, as a means of knowledge transfer, mainly by recreating educational contents as engaging and entertaining educational experiences can be created in VR. More specifically, it is very effective for learning contexts such as anatomy, geography, history, arts, exploration and building models of the world. It can also allow learners to take part in roleplay exercises in social group interaction and, as such, fosters knowledge retention, minimizes the risk of laboratory accidents, promotes distant learning, and helps assess the quality of acquired knowledge via different types of techniques (Burdea and Coiffet, 2003). For example, in educational environments, multisensory stimulation similar to what VR can offer today is found to foster meaningful learning in elementary students since it supports concepts linked to mathematics, visualization, and spatial navigation linking them to multisensory association (Cuturi et al., 2022).

When discussing VR applications in learning environments, it is important to reflect on the role of the senses, since they are the main inputs of information while learning, and this should be no different in virtual environments. The senses can be framed as body mechanisms that collect information of what is happening inside and outside of the individual, or the surrounding environment (Barlow, 1982). When assessing the range of multisensory stimulation in learning environments, it is evident that the current consensus around the world is that humans have different senses with which to interact with both the external and the internal world (Scerri et al., 2021). Although there could be more categories of senses, most literature group the senses in sight, smell, taste, touch, and hearing.

All senses are connected to the brain and the way the brain perceives and constructs reality is mostly via a combination of information coming from the range of senses. Such information —that can be either physical or digital—combined in a multisensory context, has been essential for human evolution and survival (Chialvo, 2006; Wang et al., 2021). As such, humans can be described as sensing beings living in a multisensory world that is constantly sensorially stimulated (Pagliano, 2012). Senses can be stimulated by elements pertaining to the natural physical world but also by stimuli carefully crafted by other humans to obtain specific results. For example, think of improving a person’s performance by, say, prompting a calm and relaxing state of mind, as in the case of some therapeutic intervention for the treatment of dementia (e.g., Haegele and Porretta, 2014; Scerri et al., 2021), or to aid post-stroke rehabilitation programs to help a patient’s recovery (Bolognini and Vallar, 2020).

Having framed VR, and the senses, in learning experiences, we move toward the theoretical basis for using VR for learning, which is provided by the Cognitive Theory of Multimedia Learning (CTML; Mayer, 2005). Such a theory actually builds on prior research from the multisensory learning field (Cleland and Clark, 1966). The CTML is based on three cognitive science principles of learning: (i) the human information processes mechanism includes dual channels for visual and auditory processing, (ii) each channel has a limited capacity for processing, (iii) and active learning involves a coordinated set of cognitive processes during learning (Mayer, 2005). Given that the CTML focuses primarily on visual and olfactory stimuli, it identifies five cognitive processes on which learning contents shall be designed, emphasizing the role of the pictorial and verbal content of the stimuli. The implication is that instructors must design multimedia messages purposefully and carefully in order to manage the cognitive load that the human brain is prone to experience in the process of learning through pictorial and verbal stimuli.

In this sense, learning can be measured either by testing the retention, that is, recall of the information presented, or by the ability to transfer the information in creative ways, such as being able to use the information to solve new problems and propose new ideas (Mayer, 2005). Recall, defined as the ability to reproduce or recognize the material presented can be assessed by two types of retention tests: (1) Recall tests, where learners are asked to reproduce what was presented, and/or (2) Recognition tests, where learners are asked to select what was presented (Smith et al., 2016; Gomes et al., 2019). Transfer, defined as the ability to construct a coherent mental representation from the presented material, which can be reflected in the ability to use the presented material in a novel situation through creative expressions, can be assessed using transfer tests such as those that ask learners to solve problems that were not given in the material presented. That is, transfer tests relate to the quality of learning (Mayer, 2005).

Summarizing, VR is a general-purpose technology that opens new avenues for expanding the Cognitive Theory of Multimedia Learning toward the inclusion of other sensory stimuli to assess the learning experience and learning outcomes related to recall and transfer (creativity), going beyond traditional visual and auditory stimulation. Moreover, the boom of content creation designed to be experienced in VR offers a broad horizon for exploration with training and learning objectives in mind despite the limitations of early experimental designs (PWC, 2020). The rapid pace of technology improvement, together with the promise to include additional sensory stimuli such as olfaction and touch in the VR experience, makes it even more attractive for research exploration in multimedia learning.

### 1.2. Research questions and testable hypotheses

Based on the aforementioned literature, we conjecture that the multisensory stimulation instrumentalized via audiovisual VR and an additional sensory stimulus (touch, smell or taste), in a stereotypical learning context, has a positive effect on the learning experience, and the learning outcomes related to recall and creativity. More specifically, we consider the existing gap in the literature concerning that smell is underexplored in multisensory VR experiences (Melo et al., 2020). Hence, as key novelty of this study, we take the case of olfactory stimulation and, consistent with the predictions of the CTML (Mayer, 2005), argue that an educational experience could be enhanced, and learning outcomes could be fostered, by immersing subjects into the multisensory world of VR and scent stimulation in a stereotypical learning environment. We hypothesize that the inclusion of a congruent scent during the teaching content would prompt higher levels of recall and creativity as well as strong emotional and motivational responses to be reflected in the degree of immersion, engagement, and entertainment.

Our sense of smell is directly connected to the most primitive cortex of the brain (Herrick, 1933), meaning that it was the first—or the most primitive—sense to be developed in humans, and most animals (Sebastian and Puranik, 2016; Shulman, 2018). Such a connection with the brain is also very fast (Wojciechowska et al., 2017), and has a strong association with recall performance (Çeven and Belkayali, 2021), providing support for immediate emotional and motivational responses (Herz, 2010). Thus, we expect that pairing VR embedded visual and auditory stimuli with scent, will create a congruent association that could enrich the educational experience, while fostering recall and understanding.

We propose the following research questions and subsequent hypotheses to be tested:

**QUESTION 1** – Which attributes of the educational experience are enhanced by the multisensory stimulation instrumentalized through VR and olfactory stimuli in a stereotypical learning setting?

H1A. Multisensory stimulation (including congruent 3D video, sound and olfaction) is perceived as enhanced in terms of quality, as compared to the stereotypical 2D audiovisual experience (involving only congruent video and sound).

H1B. Multisensory stimulation (including congruent 3D video, sound and olfaction) triggers higher rates of sensations and emotions related to immersion, as compared to the stereotypical 2D audiovisual experience (only congruent 2D video and sound).

**QUESTION 2** – Does multisensory stimulation instrumentalized through VR and olfactory stimuli enhance recall in a stereotypical learning setting?

H2. Multisensory stimulation (including congruent 3D video, sound and olfaction) provides support for more effective recall, as compared to the stereotypical 2D audiovisual experience (only congruent 2D video and sound).

**QUESTION 3** – Does multisensory stimulation instrumentalized through VR and olfactory stimuli foster creativity in a stereotypical learning setting?

H3. Multisensory stimulation (including congruent 3D video, sound and olfaction) provides a more suitable environment for creative copywriting, as compared to the stereotypical 2D audiovisual experience (only congruent 2D video and sound).

## 2. Materials, data, and methods

As the augmented type of technology, for this study, we chose to work with VR goggles mounted on a smartphone, as this is much more accessible in terms of costs and availability, it is easier to use, and it allows the learner to quickly continue interacting with other physical tools that are a usual part of the stereotypical learning setting, such as a physical notebook or a PC. The audio-visual stimulus used in our study focused on one single topic: the context and process of harvesting and distillation of French lavender.

To assess the retention of the concepts presented in the video (recall), we conducted a recognition test. Meanwhile, to assess transfer and creativity, we gave the participants the task of constructing a coherent mental representation of the process of harvesting and distillation of French lavender, and then observed whether they had the ability to use the presented material in a novel situation not disclosed prior to and during the experience. The way the participants were asked to use such new mental representation was by instructing them to create a short sentence, similar to a tweet, using the new knowledge acquired, to attract tourists to Provence, where French lavender is harvested and distilled in the video.

As the scent stimulus, we used a physical version of lavender scent together with the audiovisual VR experience. Here, we thought that the sense of smell would add a congruent semantic layer to the content of the visual and auditory stimuli, which are about French lavender production (as in semantic congruence).

Importantly, and besides assessing the above-mentioned learning outcomes, the objective of immersing the participants in a multisensory world was also to observe whether they could perceive the taught material as more immersive, engaging, and entertaining.

### 2.1. Participants

One hundred and ninety participants were included in the experiment. They were recruited around Universidad de los Andes campus, located in downtown Bogotá, capital of Colombia. Participants were homogenous in gender (56.84% females), aged between 18 and 24 years, with a mean of age of 20 years (SD = 2). All participants were students enrolled in the University, and from diverse academic undergraduate programs ranging from first to fifth semesters. The majority were single or expressed that they did not live with a spouse or partner and came from middle and upper social economic classes according to Colombian social standards.

### 2.2. Stimuli

#### 2.2.1. Video

A video with voiceover in Spanish, of approximately 6 min and 40 s, was created focusing on lavender production, with the content in the following chronological order: (a) broad general facts about France, (b) culturally important symbols of France such as the Louvre Museum and the Eiffel Tower among others, (c) general facts about the region of Provence, (d) the importance of lavender fields in Provence, (e) the process of lavender harvesting and collection, (f) the chemical process of lavender distillation to make lavender-based-essence for the perfume industry. The video was edited and converted into traditional 2D, and in 3D format, the latter to be seen using VR goggles for smartphones.

All participants who were exposed to the VR video used a pair of VR glasses/goggles and used a smartphone that had an app to visualize VR Videos (for more details, please see Figures S1, S2, in the Supplementary material, which provide examples of the experiment setup and tools).

The 2D and 3D videos can be accessed here:

https://youtu.be/1Zbp00hPzCE

https://youtu.be/lOFCNpCehJA

#### 2.2.2. Fragrance

A sample of French lavender essential oil provided by My Zent sensory marketing company was used for this study as an olfactory stimulus.^1^ All the participants who were exposed to this scent received a strip of paper imbued with an oil-based essence, and they had to smell it when they were asked to do so in the video. Two drops of French lavender essence were dropped on each strip and these were then set aside for 30 min. After 30 min, the strips were used for a maximum of 60 min. Once the 60 min had elapsed, all the strips that were not used were discarded and new strips were activated using the same protocol to ensure an homogeneus level of olfactory stimulus intensity range.

### 2.3. Experimental setup and design

The randomized experiment was conducted on Universidad de los Andes premises. Data collection occurred from October 25th to 29th, 2021, between 10:00 a.m. and 2:00 p.m. Each participant that was invited and accepted to join the experiment, had to read an initial informed consent. Once they consented, they had access to a communal desk with eight experiment booths working at the same time. Each participant had a chair, a computer screen to watch the video, a pair of Sennheiser HD206 Over Ear Headphones, an Intel sound card at 50% volume, access to Youtube platform where the video was shown at 100% volume range. While conducting the experiment, the participants could not interact with each other. Moreover, the distance between individual booths was enough to ensure that participants did not see each other’s screens, nor smell other students’ olfactory stimulus.

Once they joined the experiment, the participants were randomly assigned into the three treatment groups or the control group. In the control group (*n* = 45) participants were exposed to a 2D video without being exposed to lavender olfactory stimulation. The participants of the first treatment group were exposed to the same 2D video but with lavender scent olfactory stimulation; namely 2D SMELL (*n* = 52). As a second treatment group, participants were exposed to a 3D video using VR goggles without lavender olfactory stimulation; namely VR (*n* = 51). Finally, the third treatment group of participants were exposed to a 3D video using VR goggles and were stimulated with lavender scent; namely VR SMELL (*n* = 42). See Table 1 for a summary of the four experimental conditions.

**Table 1:** Summary of the four experimental conditions.

Treatment	Participants (*n*)	Stimulus
Control (2D)	45	2D video without lavender olfactory stimulation
2D SMELL	52	2D video with lavender scent olfactory stimulation
3D	51	3D video using VR goggles without lavender olfactory stimulation
3D SMELL	42	3D video using VR goggles with lavender olfactory stimulation

Once they had watched the video, participants were asked to access the Qualtrics platform^2^ with an electronic questionnaire presented in Spanish consisting of four main blocks, plus demographics, which lasted no more than 10 min to complete. In the first part, participants answered nine 7-point Likert scale questions concerning their general perception of the learning experience (*quality of audiovisual production, quality of content, comfort while watching, clarity of content, level of distraction, sense of video, whether they liked the video, comprehension, attention*).

In the second part, the participants answered ten 7-point Likert scale questions concerning sensations and emotions arising from the immersion associated with the proposed learning experience (*important, boring, striking, unnecessary, exciting, useful, attractive, relevant, insignificant, full of sensations*).

The third part of the questionnaire was used to assess recall. Here, participants answered five multiple-choice questions, where they had to choose one correct answer concerning the content they watched *(What is the first step in lavender essence production?; What is the last step in lavender essence production?; Which of France’s cultural symbols was shown first in the video?; Which of France’s cultural symbols was shown second in the video? Why is Grasse considered the perfume capital of the world?)*.

The fourth section of the questionnaire was used to assess creativity. Here, participants had to create a short tweet that could potentially be used in social media. The task was as follows: *The city of Grasse, the world capital of perfume, is located in the region of Provence. Its fields of lavender, jasmine, mimosa, and rose and the ideal microclimate for these flower crops, used to attract thousands of tourists each year. However, following the COVID-19 pandemic, the number of tourists visiting the city has dropped significantly. How can Grasse attract more tourists? Consider that you have been designated to help solve this problem in a creative way, using Twitter as a medium. Your mission is to create a promotional tweet, to help the city attract more tourists.*

The last part of the questionnaire contained basic demographic questions. The order of sections, and the questions within each section of the questionnaire, were presented randomly. The order of the multiple-choice answers was also randomized. This experimental protocol was revised and approved by the Universidad de los Andes, School of Management Ethics Committee (memo n.75, of the 26th of October, 2020).

### 2.4. Data collection and analysis

Data from all participants was exported from Qualtrics in Excel tables. The first verification of normality across the data failed. Hence, a heteroscedasticity analysis was performed using Levene’s test, to verify equal variances between groups (homoscedasticity;^3^ Phillips, 1995). Since the data did not meet this condition either, a general correction was applied to the entire dataset. This correction was based on calculating the standard deviation of each treatment group and then dividing each data point of each treatment group by the standard deviation of the treatment group to which it belonged (Phillips, 1995). Once this correction was applied, the Levene test was repeated, and the condition of homoscedasticity was met.

For the first and second sections of the survey (Likert-scales data), two independent multivariate analyses of variance were conducted, with the treatments as between-participants factor, and the questions as dependent variables. Pairwise comparisons were Tukey corrected.

For the third section of the survey (multiple-choice based), one analysis of variance was conducted, with the treatment as between-participants factor. Here, a new dependent variable was created (namely *recall*), which consisted of the sum of the responses of each participant (e.g., “1” if the response was correct and “0” if the response was incorrect). Pairwise comparisons were Tukey corrected.

For the fourth section of the survey (Tweet question), the cosine similarity output from Qualtrics was chosen to assess such data. Cosine similarity measures represent the similarity between two vectors of an inner product space. It is often used to measure document similarity in text analysis (Han et al., 2022). Hence, we used this measure to compare and analyze the level of similarity of the short tweets created by the participants, in each treatment, thus creating a proxy for uniqueness. Briefly, a higher cosine indicated that the texts prepared by the participants in a certain condition were more similar in terms of wording and, hence, showing lower creativity. A lower cosine indicated that the texts prepared by the participants in a certain condition were more distinctive in terms of wording (i.e., higher usage of unique words) and, thus, more creative. The cosine data was subjected to an analysis of variance, with the treatments as between-participants factor. Pairwise comparisons were Tukey corrected. The significance of all the quantitative analyses conducted in this study was set with a 95% confidence.

Several qualitative analyses were also conducted using the fourth section of the survey (i.e., word-cloud, word correlations, comparison graphics, deductive-oriented analysis). However, the results obtained from such a qualitative perspective did not bring insights beyond the conclusions brought by the cosine similarity assessment. Therefore, these analyses are not included as part of the following results.

## 3. Results

### 3.1. General perceived quality of the learning experience

Both multivariate main effects and multivariate interaction were statistically significant (Pillai’s trace = 26.907; *p*-value = <0.001; η^2^ = 0.574). The Univariate test showed that the variables *attention, attractiveness, quality of content, sense of video, comfort while watching, clarity of content, comprehension,* and *quality of audiovisual production* received significantly different scores between treatment groups (*p* ≤ 0.05; see Table 2 for detailed results). *Post hoc* analysis shows that the 2D SMELL treatment group scores were significantly higher in terms of *attention* (compared to VR), *comfort while watching* (compared to control, VR and VR SMELL)*, clarity of content* (compared to control, VR and VR SMELL)*, comprehension* (compared to control, VR and VR SMELL)*, quality of content* (compared to control and VR)*, quality of audiovisual production* (compared to control, VR and VR SMELL), and *sense of video* (compared to VR). *Post hoc* analysis also shows that scores of the control group (2D) were higher in terms of *attention* (compared to VR)*, comfort (compared to VR and VR SMELL), clarity of content* (compared to VR) and *quality of audiovisual production* (compared to VR and VR SMELL). VR SMELL scored higher in terms *of clarity of content* (compared to control and VR)*, comprehension* (compared to control and VR)*, quality of audiovisual production* (compared to control) and *quality of content* (compared to control and VR) (*p* ≤ 0.05; see Table 2).

**Table 2:** Univariate—left—and *post-hoc*/pairwise comparisons—right—analyses on general perceived quality of the video experience.

Dependent variable	Group (i)	*F*	*p*	η^2^	Pairwise comparison (j) Mean difference (i–j)			
					Control	2D SMELL	VR	VR SMELL
Attention	Control	4.813*	0.003	0.072	–	−0.129 (0.921)	0.578* (0.026)	0.175 (0.847)
	2D SMELL				0.129 (0.921)	–	0.708* (0.002)	0.304 (0.460)
	VR				−0.578* (0.026)	−0.708* (0.002)	–	−0.404 (0.216)
	VR SMELL				−0.175 (0.847)	−0.304 (0.460)	0.404 (0.216)	–
Comfort while watching	Control	95.634*	<0.001	0.607	–	−2.279* (<0.001)	0.640* (0.011)	0.604* (0.028)
	2D SMELL				2.279* (<0.001)	–	2.918* (<0.001)	2.882* (<0.001)
	VR				−0.640* (0.011)	−2.918* (<0.001)	–	−0.0358 (0.998)
	VR SMELL				−0.604* (0.028)	−2.882* (<0.001)	0.0358 (0.998)	–
Clarity of content	Control	127.544*	<0.001	0.673	–	−2.686* (<0.001)	1.043* (<0.001)	−0.867* (<0.001)
	2D SMELL				2.686* (<0.001)	–	3.729* (<0.001)	1.818* (<0.001)
	VR				−1.043* (<0.001)	−3.729* (<0.001)	–	−1.910* (<0.001)
	VR SMELL				0.867* (<0.001)	−1.818* (<0.001)	1.910* (<0.001)	–
Comprehension	Control	65.898*	<0.001	0.515	–	−2.140* (<0.001)	0.258 (0.588)	−1.469* (<0.001)
	2D SMELL				2.140* (<0.001)	–	2.398* (<0.001)	0.671* (0.008)
	VR				−0.258 (0.588)	−2.398* (<0.001)	–	−1.727* (<0.001)
	VR SMELL				1.469* (<0.001)	−0.671* (0.008)	1.727* (<0.001)	–
Sense of video	Control	3.367*	0.020	0.052	–	−0.487 (0.082)	0.0501 (0.995)	0.0331 (0.999)
	2D SMELL				0.487 (0.082)	–	0.537* (0.035)	0.520 (0.062)
	VR				−0.0501 (0.995)	−0.537* (0.035)	–	−0.0175 (1.000)
	VR SMELL				−0.0331 (0.999)	−0.520 (0.062)	0.0175 (1.000)	–
Likeness	Control	54.245*	<0.001	0.467	–	−1.493 (<0.001)	0.175 (0.827)	−1.999 (<0.001)
	2D SMELL				1.493 (<0.001)	–	1.668 (<0.001)	−0.507 (0.073)
	VR				−0.175 (0.827)	−1.668 (<0.001)	–	−2.175 (<0.001)
	VR SMELL				1.999 (<0.001)	0.507 (0.073)	2.175 (<0.001)	–
Quality of content	Control	55.088*	<0.001	0.470	–	−1.760* (<0.001)	−0.309 (0.434)	−2.260* (<0.001)
	2D SMELL				1.760* (<0.001)	–	1.451* (<0.001)	−0.499 (0.079)
	VR				0.309 (0.434)	−1.451* (<0.001)	–	−1.951* (<0.001)
	VR SMELL				2.260* (<0.001)	0.499 (0.079)	1.951* (<0.001)	–
Quality of audiovisual production	Control	92.062*	<0.001	0.598	–	−1.577* (<0.001)	1.310* (<0.001)	1.262* (<0.001)
	2D SMELL				1.577* (<0.001)	–	2.887* (<0.001)	2.839* (<0.001)
	VR				−1.310* (<0.001)	−2.887* (<0.001)	–	−0.048 (0.996)
	VR SMELL				−1.262* (<0.001)	−2.839* (<0.001)	0.048 (0.996)	–

*indicate a significant difference at 95% confidence (*p* < 0.5).

### 3.2. Sensations and emotions analysis

Both multivariate main effects and the multivariate interaction were statistically significant (Pillai’s trace = 10.373; *p*-value = <0.001; η^2^ = 0.367). The Univariate test showed that the variables: *striking, relevant, full of sensations, important, exciting, unnecessary,* and *attractive* received significantly different scores between conditions (*p* ≤ 0.05; see Table 3 for detailed results). *Post hoc* analysis showed that the 2D SMELL scores were significantly higher in terms of *striking* (compared to control, VR and VR SMELL)*, relevant* (compared to control), *full of sensations* (compared to control and VR), *exciting* (compared to control and VR)*, unnecessary* (compared to control and VR), and *attractive* (compared to control, VR and VR SMELL). On the other hand, the VR SMELL treatment group scores were significantly higher for *striking* (compared to control, 2D SMELL, and VR), *full of sensations* (compared to control and VR) *important* (compared to control and VR), *exciting* (compared to control), and *unnecessary* (compared to control) (*p* ≤ 0.05; see Table 3).

**Table 3:** Univariate—left—and *post-hoc*/pairwise comparisons—right—analyses concerning sensations and emotion scores evoked by the video experience.

Dependent variable	Group (i)	*F*	*p*	η^2^	Pairwise comparison (j) Mean difference (i–j)			
					Control	2D SMELL	VR	VR SMELL
Striking	Control	55.957*	<0.001	0.474	–	−2.524* (<0.001)	−0.7696* (0.001)	−1.445* (<0.001)
	2D SMELL				2.524* (<0.001)	–	1.754* (<0.001)	1.079* (<0.001)
	VR				0.7696* (0.001)	−1.7544* (<0.001)	–	−0.676* (0.008)
	VR SMELL				1.445* (<0.001)	−1.079* (<0.001)	0.676* (0.008)	–
Relevant	Control	5.718*	<0.001	0.084	–	−0.821* (<0.001)	−0.325 (0.388)	−0.516 (0.079)
	2D SMELL				0.821* (<0.001)	–	0.496 (0.060)	0.305 (0.457)
	VR				0.325 (0.388)	−0.496 (0.060)	–	−0.191 (0.795)
	VR SMELL				0.5161 (0.079)	−0.305 (0.457)	0.191 (0.795)	–
Full of sensations	Control	43.104*	<0.001	0.410	–	−1.398* (<0.001)	0.073 (0.985)	−1.815* (<0.001)
	2D SMELL				1.398* (<0.001)	–	1.471* (<0.001)	−0.417 (0.188)
	VR				−0.073 (0.985)	−1.471* (<0.001)	–	−1.888* (<0.001)
	VR SMELL				1.815* (<0.001)	0.417 (0.188)	1.888* (<0.001)	–
Important	Control	3.58*	0.015	0.055	–	−0.306 (0.438)	−0.099 (0.963)	−0.646* (0.015)
	2D SMELL				0.306 (0.438)	–	0.207 (0.720)	−0.341 (0.358)
	VR				0.099 (0.963)	−0.207 (0.720)	–	−0.547* (0.046)
	VR SMELL				0.646* (0.015)	0.341 (0.358)	0.547* (0.046)	–
Exciting	Control	10.541*	<0.001	0.145	–	−1.072* (<0.001)	−0.468 (0.105)	−0.865* (<0.001)
	2S SMELL				1.072* (<0.001)	–	0.604* (0.013)	0.207 (0.752)
	VR				0.468 (0.105)	0.604* (0.013)	–	−0.398 (0.228)
	VR SMELL				0.865* (<0.001)	−0.207 (0.752)	0.398 (0.228)	–
Unnecessary	Control	4.03*	0.008	0.061	–	−0.662* (0.007)	−0.145 (0.894)	−0.251 (0.646)
	2D SMELL				0.662* (0.007)	–	0.517* (0.046)	0.411 (0.199)
	VR				0.145 (0.894)	−0.517* (0.046)	–	−0.106 (0.957)
	VR SMELL				0.251 (0.646)	−0.411 (0.199)	0.106 (0.957)	–
Attractive	Control	35.61*	<0.001	0.365	–	−1.916* (<0.001)	−0.503 (0.070)	−0.318 (0.449)
	2D SMELL				1.916* (<0.001)	–	1.412* (<0.001)	1.597* (<0.001)
	VR				0.503 (0.070)	−1.412* (<0.001)	–	0.185 (0.812)
	VR SMELL				0.318 (0.449)	−1.597* (<0.001)	−0.185 (0.812)	–

*indicate a significant difference at 95% confidence (*p* < 0.5).

### 3.3. Recall performance analysis

Table 4 shows statistically significant differences between conditions (*F* value = 5.141; *p*-value = 0.002; η^2^ = 0.077). Concerning the pairwise comparisons, the recall results related to the scores of the control group were significantly higher when compared to the VR SMELL recall data. The scores of the 2D SMELL group were also significantly higher when compared to the VR SMELL (*p* ≤ 0.05). The rest of the comparisons did not show statistically significant differences.

**Table 4:** Recall Performance results.

Dependent variable	Group (i)	*F*	*p*	η^2^	Pairwise comparison (j) Mean difference (i-j)			
					Control	2D SMELL	VR	VR SMELL
Recall	Control	5,141*	0.002	0.077	–	0.0451 (0.996)	0.4613 (0.112)	0.699* (0.007)
	2D SMELL				−0.0451 (0.996)	–	0.4161 (0.153)	0.6538* (0.010)
	VR				−0.4613 (0.112)	−0.4161 (0.153)	–	0.2377 (0.665)
	VR SMELL				−0.699* (0.007)	−0.6538* (0.010)	−0.2377 (0.665)	–

Left side of the table shows the main test of the analysis of variance, whereas the right side of the table reports the *post-hoc* pairwise comparisons.

*indicate a significant difference at 95% confidence (*p* < 0.5).

### 3.4. Creativity analysis

Table 5 shows that the test was statistically significant (*F* value = 6.930; *p*-value <0.001; η^2^ = 0.101). In particular, the control group Cosine similarity index scored significantly higher than both VR treatments. 2D SMELL reported no significant differences between treatments.

**Table 5:** Cosine similarity results.

Dependent variable	Group (i)	*F*	*p*	η^2^	Pairwise comparison (j) Mean difference (i–j)			
					Control	2D SMELL	VR	VR SMELL
Cosine similarity	Control	6.93*	<0.001	0.101	–	0.3822 (0.241)	0.8522* (<0.001)	0.7436* (0.004)
	2D SMELL				−0.3822 (0.241)	–	0.4701 (0.083)	0.3614 (0.305)
	VR				−0.8522* (<0.001)	−0.4701 (0.083)	–	−0.1087 (0.954)
	VR SMELL				−0.7436* (0.004)	−0.3614 (0.305)	0.1087 (0.954)	–

Left side of the table shows the main results, whereas the right side of the table shows the post-hoc analysis.

*indicate a significant difference at 95% confidence (*p* < 0.5).

### 3.5. Summary of results

The obtained results associated with the general perceived quality of the video experience in Section 3.1 showed that the 2D SMELL condition was rated as the highest in terms of quality, followed by the control group scores. VR had the lowest ratings in terms of the general perceived quality of the video experience. When it comes to sensations and emotions ratings presented in Section 3.2—again, 2D SMELL—scored the highest among most variables, followed by VR SMELL. The results concerning recall in Section 3.3 suggest that the control and 2D SMELL group participants performed better than those exposed to the VR SMELL treatment. Finally, the quantitative creativity analysis in Section 3.4 suggests that VR and VR SMELL treatments produced better results than the control group.

## 4. Discussion, limitations, implications, and future work

The goal of this study was to evaluate whether a stereotypical learning experience augmented via VR and/or olfactory stimulation would increase qualitative and immersive perceptions of learners, as well as improve their ability to remember the content and/or to provide a more suitable condition for creativity (i.e., writing a creative tweet related to the video’s content). For the stereotypical audiovisual experience, we used a 2D video created with basic professional care and streamed via a digital platform (YouTube), as such type of content has become standard educational input in, for instance, university courses in all disciplines.

The study included three treatment groups and a control group. The control group were subjected to a 2D audiovisual stimulus. The treatment groups to a 2D video augmented by a lavender scent (2D SMELL); a 360-degree VR audiovisual stimulus (VR); and a VR video stimulus augmented by a lavender scent (VR SMELL).

According to the results, Hypothesis H1A which suggests that multisensory stimulation is perceived as enhanced in terms of quality, as compared to the stereotypical 2D audiovisual experience, was partially supported. The 2D SMELL treatment had the highest ratings of perceived quality (followed by 2D). Thus, the evidence is consistent with the conjecture that the olfactory stimulation bundled with a 2D video was indeed perceived as contributing to an augmented experience. Arguably, a congruent scent, in this case, lavender, boosted perceptions associated to the quality to the overall experience (Flavián et al., 2021). In other words, during the multisensory audiovisual experience, the scent of lavender was paired with images related to the harvesting and distillation of the crop, thus reaching a semantic congruency that augmented the perceived quality of the audiovisual experience. As a result, the perceived quality was rated as higher by the participants as compared to the control group but also as compared to the other treatment groups.

The perceptions related to sensations and emotions were rated as the highest under the treatment of 2D SMELL, followed by VR SMELL. This evidence provides support to H1B, according to which multisensory stimulation triggers higher ratings of sensations and emotions related to immersion, as compared to the stereotypical 2D experience. The augmented effect provided by the congruent scent prompted a more suitable environment for participants to frame themselves as immersed into the multisensory experience, where sensations and emotions related to such an experience were perceived more substantially (i.e., *striking, full of sensations, exciting*) (Flavián et al., 2021). When comparing the different results of the VR SMELL treatment, there is an apparent contradiction between the low perception of quality reported in Section 3.1 (where VR smell had low ratings in terms of perceived quality), and the high perception of immersion reported in Section 3.2 (where VR smell had the highest ratings when it comes to sensations related to immersion, i.e., full of sensations). It is possible that the novelty of using VR goggles may have caused distraction, while significantly diminishing the perceived overall quality of the multisensory experience but not necessarily the perceived immersion. The VR system used in this study was purposely inexpensive and relied on the participant using their hands and smartphone while watching the video (see Supplementary material for visuals of the experimental set-up). Even though such a system can facilitate mass VR adoption due to simplicity and cost, it certainly requires high involvement and concentration from the user, potentially causing distraction and, consequently, undesired cognitive load, especially for those who have never used such a system before (Frederiksen et al., 2020). An alternative explanation recognizing the primordial role of scents in immersive VR experiences is also worth exploring. It is established that in exposure therapy applied for anxiety and trauma related disorders, olfactory stimuli increase presence in virtual environments (Munyan et al., 2016). Another well-known fact is that scents influence judgments (Schnall et al., 2008) and regulate behavior (Holland et al., 2005). Future work should carefully examine the role of the olfactory stimuli in educational contexts that use VR immersions because under certain conditions olfactory stimuli might work as a dominant component in immersive multisensory learning experiences.

Recall analysis suggests that participants in the control group were better at effectively performing recall and recognition tests than the VR treatment groups, with VR SMELL showing the lowest scores. Therefore, H2 was not supported. The complexity of manually dealing with the VR goggles and the olfactory stimulus may have led to extraneous processing (Mayer and Mayer, 2005), again, leading to undesired cognitive load (Sweller, 2011), which prevented participants from more effectively remembering the video content.

Analysis of transfer and creativity was performed by assessing the originality of performing a novel task. The results suggest that VR and VR SMELL were more conducive to creativity than the control group, lending some support to H3 which advances that multisensory stimulation provides a more suitable environment for creative copywriting, as compared to the stereotypical 2D experience. Arguably, and despite the complexity of manually handling the VR goggles and the olfactory stimulus, students came up with unique creative ideas when exposed to more immersive experiences. Previous studies have shown that VR experiences boost confidence (PWC, 2020), and it is known that confidence drives creative outcomes (Karwowski and Beghetto, 2019). While we were unable to pinpoint to the specific mechanisms that link immersive experiences and creativity, future work shall delve deeper into this positive relation in learning contexts.

In essence, including VR and olfactory stimuli in the stereotypical learning context can increase its perceived quality, immersion, and even foster creativity. However, if the learning objective is related to recall, the limited processing capacity of each sensory channel may lead to an overload as a result of nonoptimal multisensory stimulation, which in the case of this study was probably related to the manual handling of goggles and smartphones. This limitation of the chosen technology shall be carefully assessed and compared to the cost of a more user friendly VR tool. Nowadays we can use more advanced VR solutions, which may make it easier for the user to interact with the system, potentially creating less undesired interference and undesired cognitive load (i.e., oculus quest^4^). Additionally, the unsophisticated way in which the video, and the multisensory experience including scent, were incorporated in this study, could certainly be improved, even if this means that the ecological validity of the study may be compromised given the stereotypical learning context.^5^

Theoretically, this research draws on the insights provided by the CTML as it analyzes how learning can be affected when supported with multimedia technology. However, CTML also explicitly states that meaningful learning does not happen because of the sole use of technology. Instead, meaningful learning depends on the cognitive activity of the learner during the learning process. This implies that what matters when designing instructional messages—such as videos—is not the interaction of the learner with technology but how technology is applied to guide the learner toward a more active cognitive process, such as thinking, while exposed to a specific stimulus. Therefore, technologies such as VR can foster learning not because the learner interacts in a VR world, but because during the learner’s immersion in the VR world the learner is led to optimize his or her cognitive process. In stereotypical learning contexts, such a condition regarding the design of the teaching content is rarely met and thus we do not claim that the present study provides a direct test of the CTML in the strictest sense. Alternatively, our hypotheses are consistent with the CTML and draw on its predictions, while adding a congruent sensory stimulus (scent), accepting the fact that the stereotypical learning context does not, as a rule, rely on multisensory immersions that were purposefully designed to optimize the cognitive activity of the learner.

Given the explosion of virtual reality technologies and content, olfactory stimulation could plausibly be added to the sounds, words, and pictures of the CTML audiovisual experience to help foster learning. Moreover, in a stereotypical audiovisual experience, VR and olfactory stimulation can bring learners to a state of immersion that is not comparable to any previously available technological solution. As such, they offer a fertile opportunity for researchers to explore the boundary conditions of multiple theories of learning. It is remarkable that the empirical evidence found for a stereotypical learning environment is consistent with most of the hypotheses drawn from the CTML and therefore affirms its relevance.

Finally, we believe that VR can turn into an upcoming mass solution for learning and, as such, it needs to be better understood by instructors and content creators. In our case, we chose to replicate a stereotypical class scenario, where instructors may not have full knowledge on how to produce a build-to-learning-objectives video, but could use a freely available online resource that touches on topics of interest. This is, in fact, the most common case, as most instructors would simply choose audiovisual content that is rarely a perfect match for some specific learning objectives. Furthermore, with the increasing adoption of VR, and if the cost of having such technology available for a large group of students decreases, the need to be practical and use a less user friendly, but very accessible goggle system, might disappear. However, until this happens, and for the purpose of more effective recall, student outcomes appear to be better with no VR system than with a complex and non-user-friendly one. As the education industry is in its very early days of understanding the full immersion of the senses as factors of human learning, future work will refine current theories of learning and push the boundaries of human capacities through technologies to limits we cannot even imagine today.

## Data availability statement

The raw data supporting the conclusions of this article will be made available by the authors upon request.

## Ethics statement

The studies involving human participants were reviewed and approved by Universidad de los Andes School of Management Ethics Committee (Memo n.75, of the 26th of October, year 2020). The participants provided their written informed consent to participate in this study.

## Author contributions

VA and FR-C conceptualized the study, developed the methodology, experimental protocol, and supervised the data collection. MJ analyzed the data and provided a report of the results. DC wrote the first draft of the manuscript. All authors contributed to the article and approved the submitted version.

## Funding

This research was funded by the Office of the vice-president of research and knowledge creation, as well as by the R&D internal funding of Universidad de los Andes School of Management.

## Conflict of interest

The authors declare that the research was conducted in the absence of any commercial or financial relationships that could be construed as a potential conflict of interest.

## Publisher’s note

All claims expressed in this article are solely those of the authors and do not necessarily represent those of their affiliated organizations, or those of the publisher, the editors and the reviewers. Any product that may be evaluated in this article, or claim that may be made by its manufacturer, is not guaranteed or endorsed by the publisher.

## Footnotes

^1^ https://myzent.com/en/

^2^ http://qualtrics.com

^3^Homoscedasticity is based on the fact that one of the conditions that must be met when performing this type of analysis is that the data must behave as if it had somewhat constant variance, as a whole. In this case, this should happen since the null hypothesis considers that all of the observations come from the same population, and thus, they should have similar average value, and steady variance.

^4^ https://www.oculus.com/experiences/quest/

^5^By ecological validity, we mean how realistic the experimental setting can be, in terms of applicability in real-life scenarios (Andrade, 2018).
