# Supplementary material for: Does multisensory stimulation with virtual reality (VR) and smell improve learning? An educational experience in recall and creativity
Source: Front Psychol. 2023 Jun 15;14:1176697. doi: 10.3389/fpsyg.2023.1176697 (PMC10308939; doi:10.3389/fpsyg.2023.1176697)
Supplement: Supplementary file 1 [file Presentation_1.pdf]

6. Supplementary Materials

Figure S1. Example of participant joining the experiment in the 2D condition (top). and in the 3D condition (bottom).

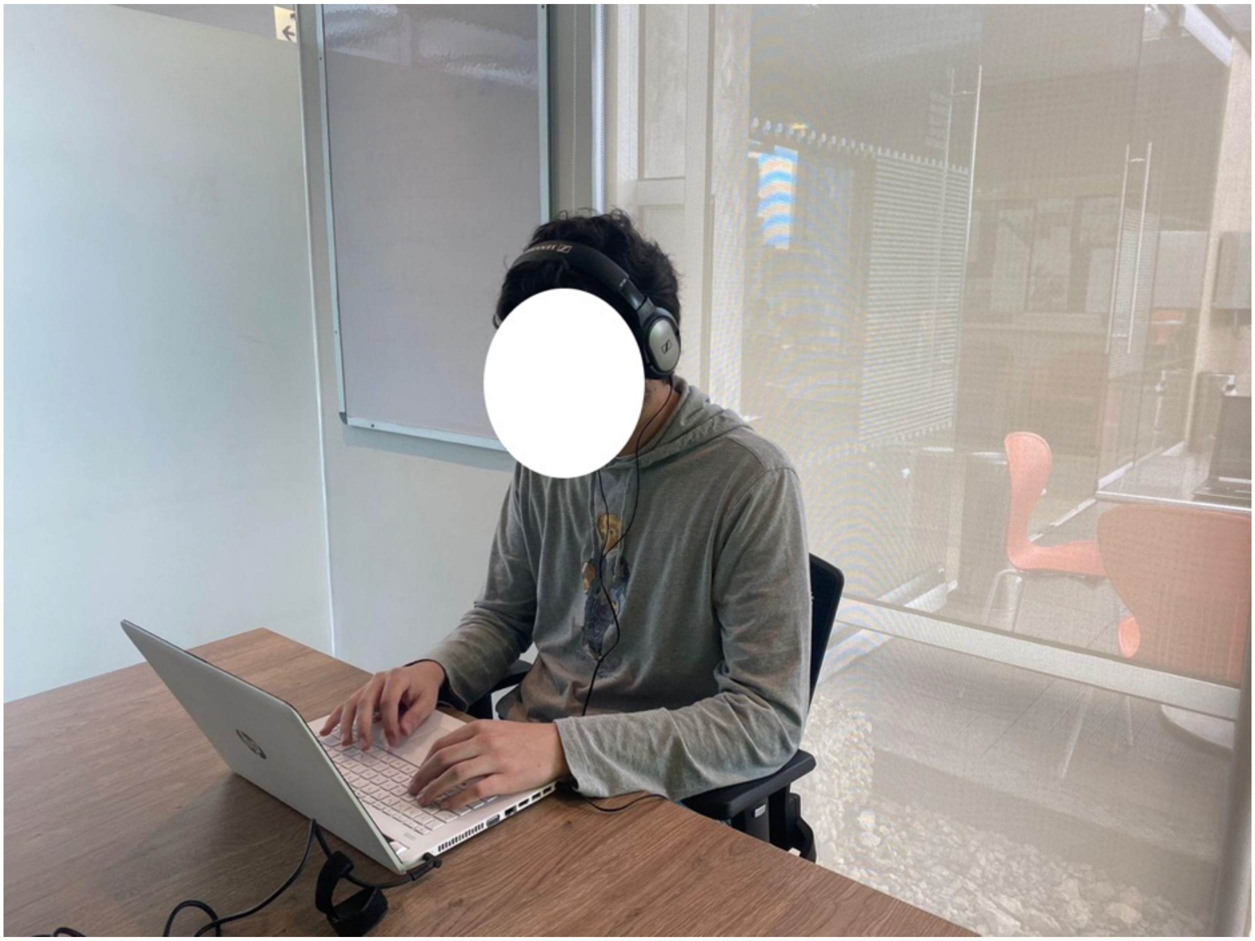

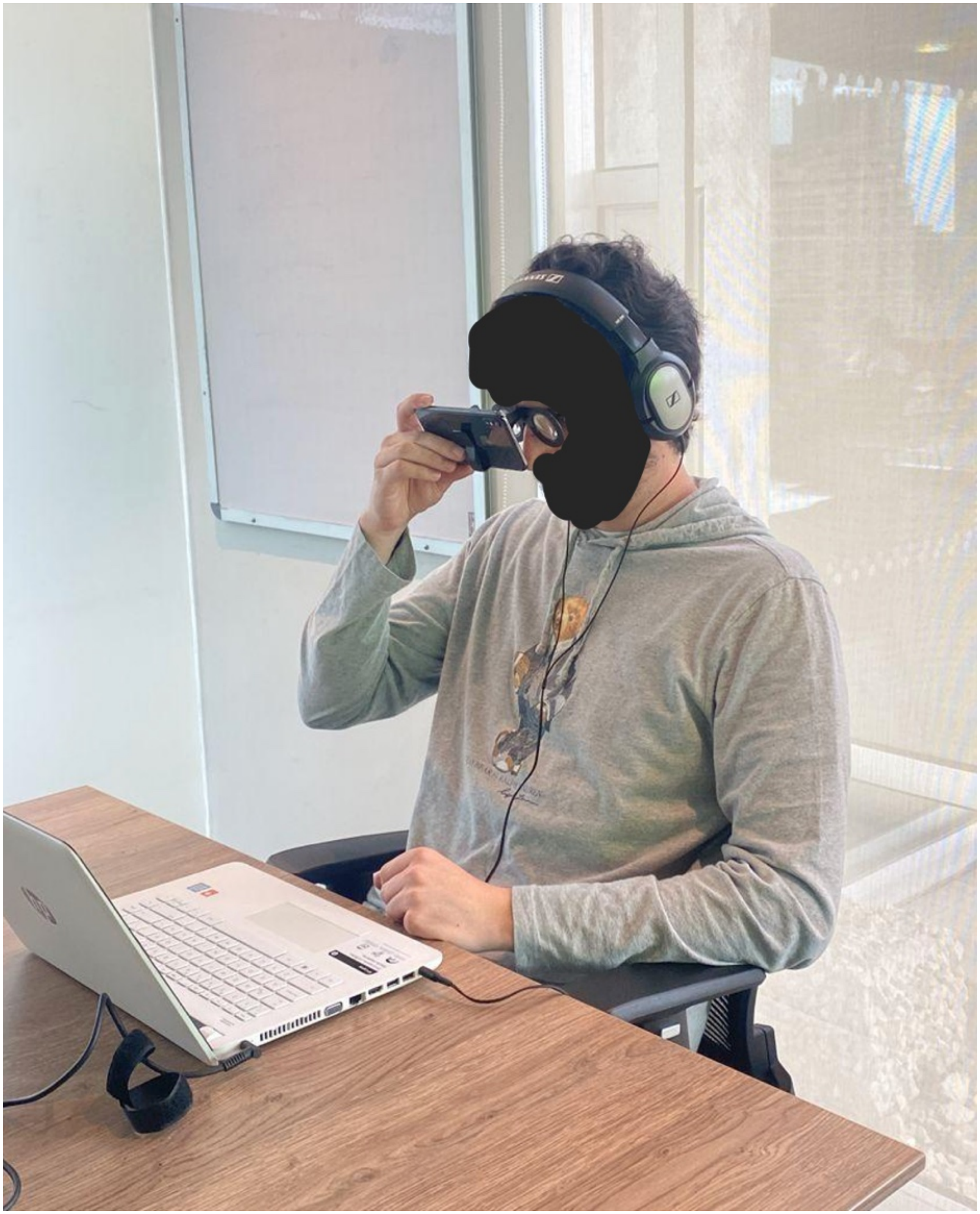

Figure S2. Pictures of the 3D goggles used in the experiment (with -top - and without -bottom - smartphone).

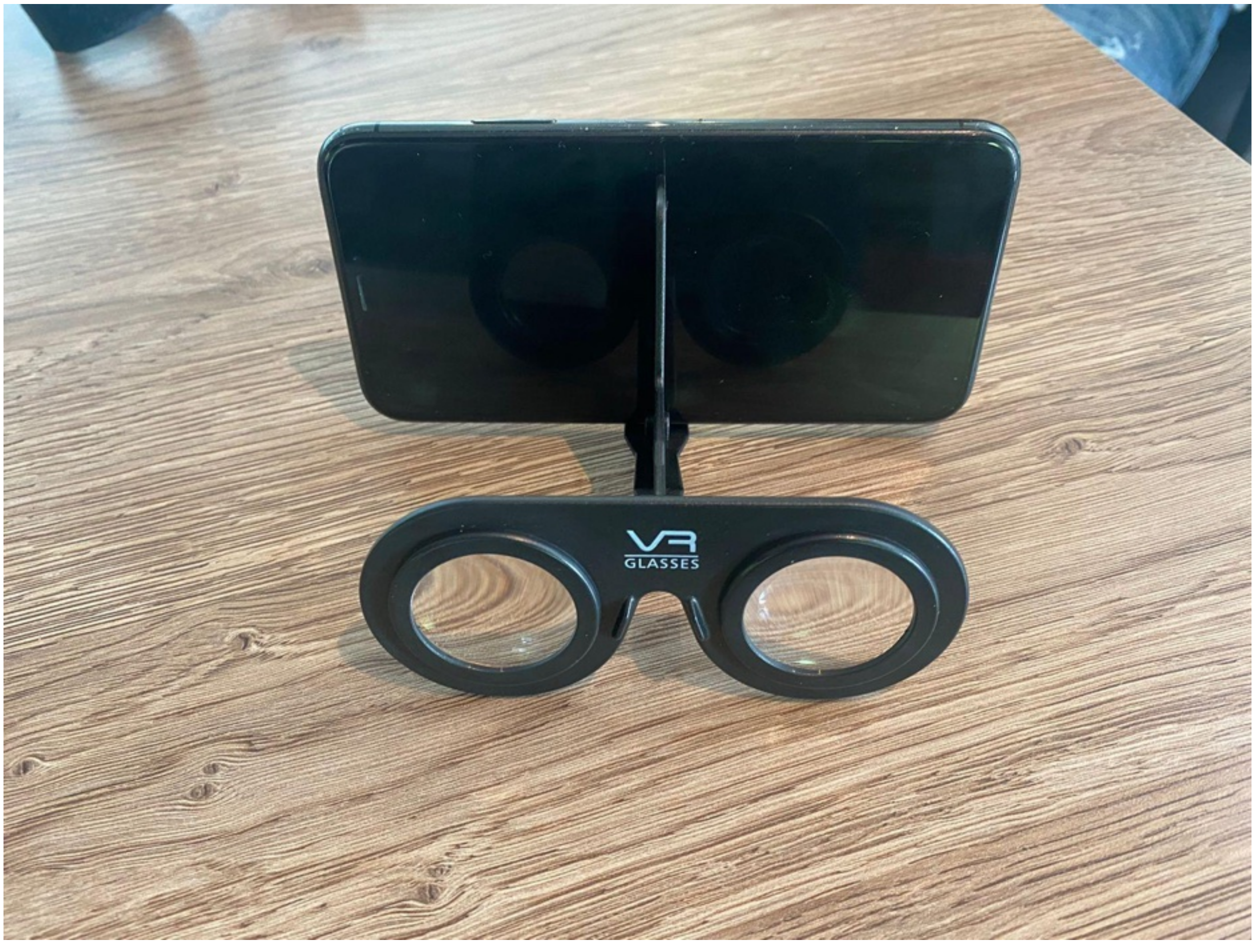

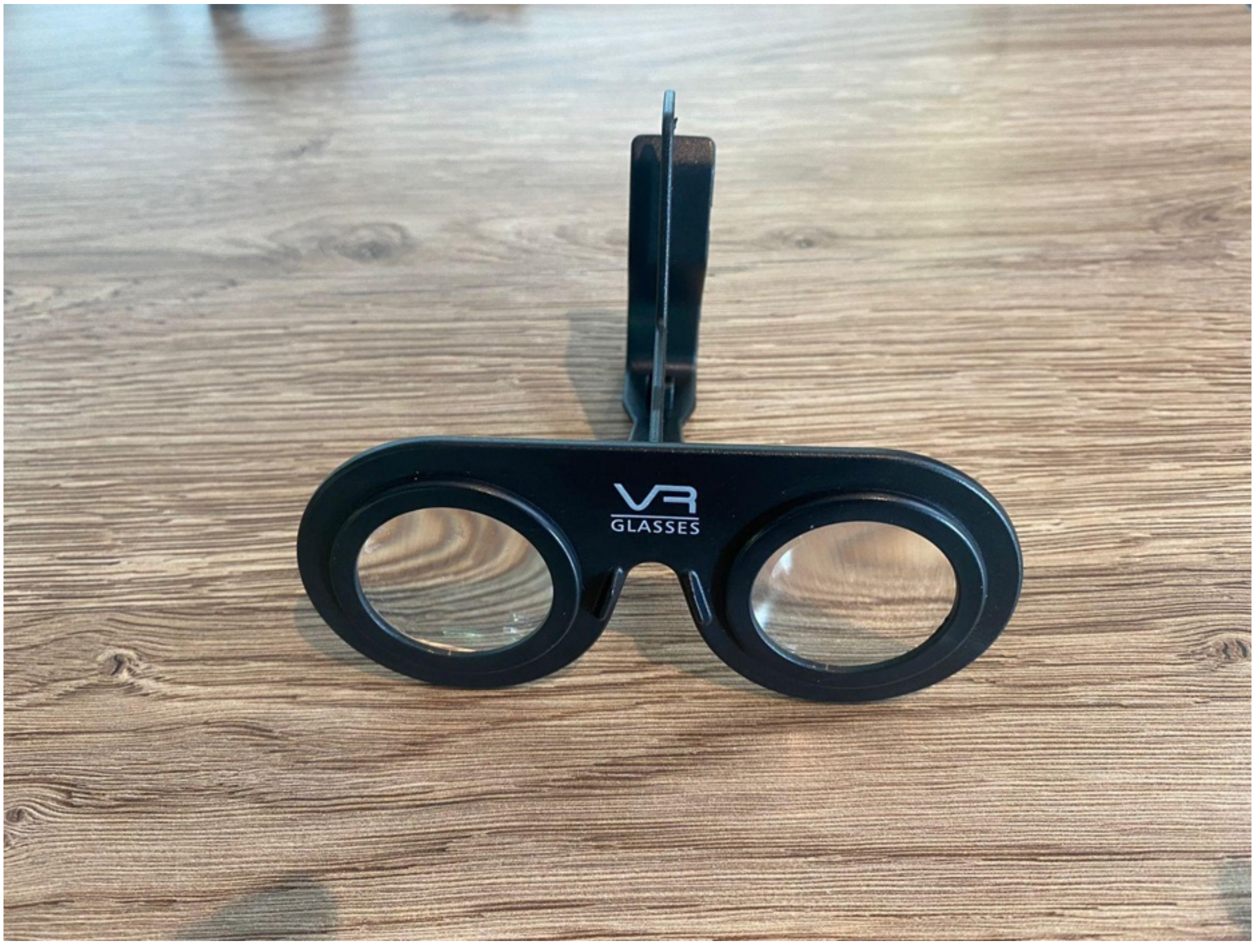
VR
GLASSES
